# Efficacy of high-energy collimator for sentinel node lymphoscintigraphy of early breast cancer patients

**DOI:** 10.2478/v10019-012-0013-3

**Published:** 2012-02-06

**Authors:** Kamran Aryana, Mohaddeseh Gholizadeh, Mehdi Momennezhad, Maryam Naji, Mohsen Aliakbarian, Mohammad Naser Forghani, Ramin Sadeghi

**Affiliations:** 1 Nuclear Medicine Research Center, Faculty of Medicine, Imam Reza Hospital, Mashhad University of Medical Sciences, Mashhad, Iran; 2 Surgical Oncology Research Center, Faculty of Medicine, Imam Reza Hospital, Mashhad University of Medical Sciences, Mashhad, Iran; 3 Cancer Research Center, Faculty of Medicine, Mashhad University of Medical Sciences, Mashhad, Iran

**Keywords:** sentinel node, lymphoscintigraphy, collimator, HEAP, high energy all purpose, low energy high resolution, LEHR

## Abstract

**Introduction:**

Lymphoscintigraphy is an important part of sentinel node mapping in breast cancer patients. Sometimes star shaped artefacts due to septal penetration can be problematic during imaging. In the current study, we evaluated the possibility of high energy (HE) collimators use for lymphoscintigraphy.

**Patients and methods:**

Twenty patients with early breast carcinoma were included. Thirty minutes after radiotracer injection (99mTc-antimony sulphide colloid), anterior and lateral images were acquired using a dual head gamma camera equipped with a parallel hole low energy high resolution (LEHR) collimator on one head and HE collimator on another head. All images were reviewed by two nuclear medicine specialists regarding detectability and number of axillary sentinel nodes and presence of star artefact.

**Results:**

All images taken by LEHR collimators showed star artefact of the injection site. No image taken by HE collimator showed this effect. In two patients the sentinel node was visible only by HE collimator. Tumour location in both of these patients was in the upper lateral quadrant and both had history of excisional biopsy. In two patients additional sentinel node was visible adjacent to the first one only on the LEHR images.

**Conclusions:**

HE collimators can be used for sentinel lymph node mapping and lymphoscintigraphy of the breast cancer patients. This collimator can almost eliminate star-shaped artefacts due to septal penetration which can be advantageous in some cases. However, to separate two adjacent sentinel nodes from each other LEHR collimators perform better.

## Introduction

Sentinel node biopsy is the standard method of axillary staging in early breast cancer patients.^1^

During surgery the sentinel nodes can be detected with two different techniques; alone or in combination: radiotracer and/or blue dye approaches.^2^ Imaging after radiotracer injection (lymphoscinitgraphy) was recommended by most guidelines which can help in performing sentinel node biopsy flawlessly.^3^ Usually after injection of the radiotracer in the breast (in a specific location according to the used protocol), lymphoscintigraphy was performed in different time intervals.^4^ A major problem while imaging the axilla, was concealment of the sentinel nodes by the activity in the injection site, scatter photons, as well as star shaped artefacts due to septal penetration.^5^,^6^ To avoid this problem many centres used a lead shield on the injection site with some success.^7^ Another approach to decrease the above mentioned problem was to use other types of collimators with thicker septa (such as medium energy) instead of usual low energy ones in order to reduce the septal penetration and decrease the star-shaped artifact.^5^–^10^ Although high energy (HE) collimators have thicker septa compared to the medium energy collimators, to the extent of our knowledge the efficacy of this kind of collimator for sentinel node mapping has not been evaluated before.

In the current study, we evaluated the feasibility and possible advantages of using HE collimator for lymphoscintigraphy of early stage breast cancer patients.

## Patients and methods

20 patients with the clinical diagnosis of early (stages I and II) breast carcinoma were included in the study. Histological diagnosis of breast cancer was based on the results of core needle biopsy or excisional biopsy.

For patients in whom the diagnosis was established by core needle biopsy periareolar intradermal injections of 18.5 MBq (0.5 mCi)/0.2 mL ^99m^Tc-antimony sulphide colloid were used. For patients with history of previous excisional biopsy of the primary lesion two intradermal injections of 18.5 MBq (0.5 mCi)/0.2 mL ^99m^Tc-antimony sulphide colloid at each end of the excisional line were given. Gentle massage was applied to the injection site subsequently for all injections for 1 minute.

Anterior and lateral views were acquired 30 minutes after the injection (2 minutes/image, 128×128 matrix, 15% energy window cantered over 140 keV) using a dual head gamma camera (e.cam Siemens), equipped with a parallel hole low energy high resolution (LEHR) collimator on one head and HE energy collimator on another head. The order of imaging was: 1) lateral view with HE, 2) lateral view with low energy, 3) anterior view with HE and 4) anterior view with low energy collimators. The outline of the patients was acquired simultaneously using the scattered photons as described by Momennezhad *et al*.^11^ The specifications of both HE and LEHR collimators are provided in Table 1. Some of the gamma camera specifications are shown in Table 2.

Collimator performance was calculated using especial calculator provided by Nuclear Fields Company.^12^

All images were reviewed by two nuclear medicine specialists regarding: detectability of axillary sentinel nodes, number of visualized sentinel nodes, and presence of star artefact. Semi-quantitative evaluation was also performed using ROIs on the injection site and on detected axillary sentinel nodes.

Quantitative data (count rates) were expressed as mean ± SD. For comparison of these quantitative data between two sets of images paired sample t tests was used. P values less than 0.05 were considered statistically significant.

The study was carried out according to the Declaration of Helsinki.

## Results

**FIGURE 1:**
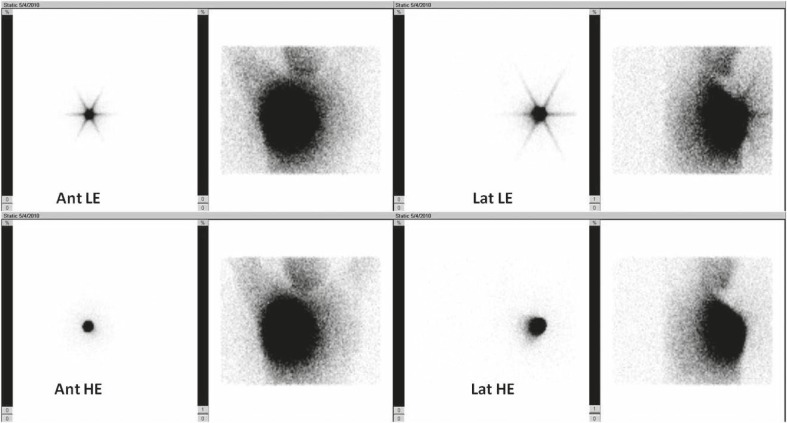
Low energy high resolution collimator (LEHR) (upper row) as well as high energy (HE) collimator (lower row) images of a patient. Scatterograms are also shown on the right sides of each original image. There is no star shape artefact in the HE collimator images.

**FIGURE 2:**
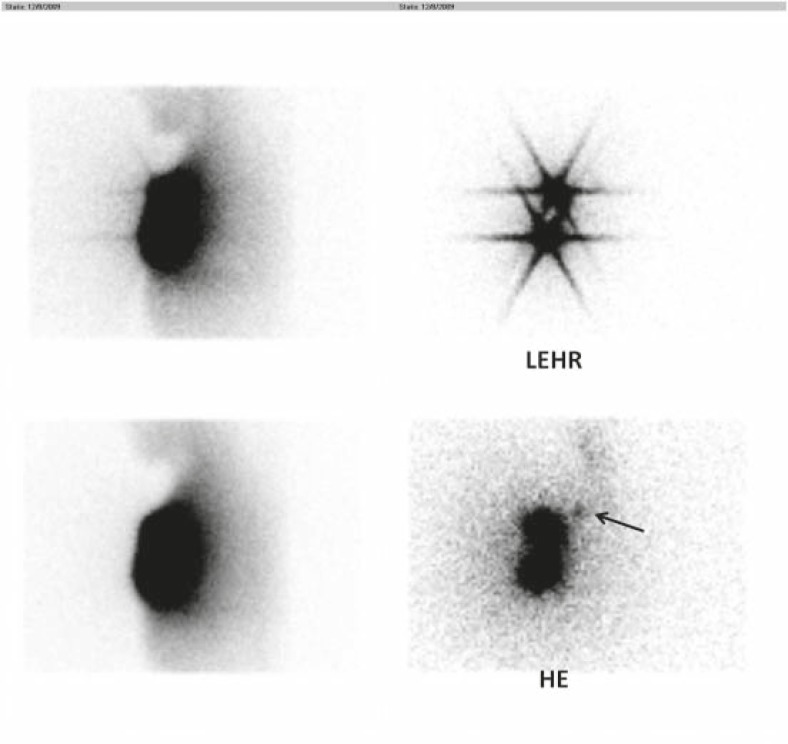
Low energy high resolution collimator (LEHR) (upper row) as well as high energy (HE) collimator (lower row) images of a patient. Note that the sentinel node is only visible on the HE collimator images (arrow) due to star artefact on the low energy collimator imaging. The scatterograms are also shown on the left side of each original image.

**FIGURE 3:**
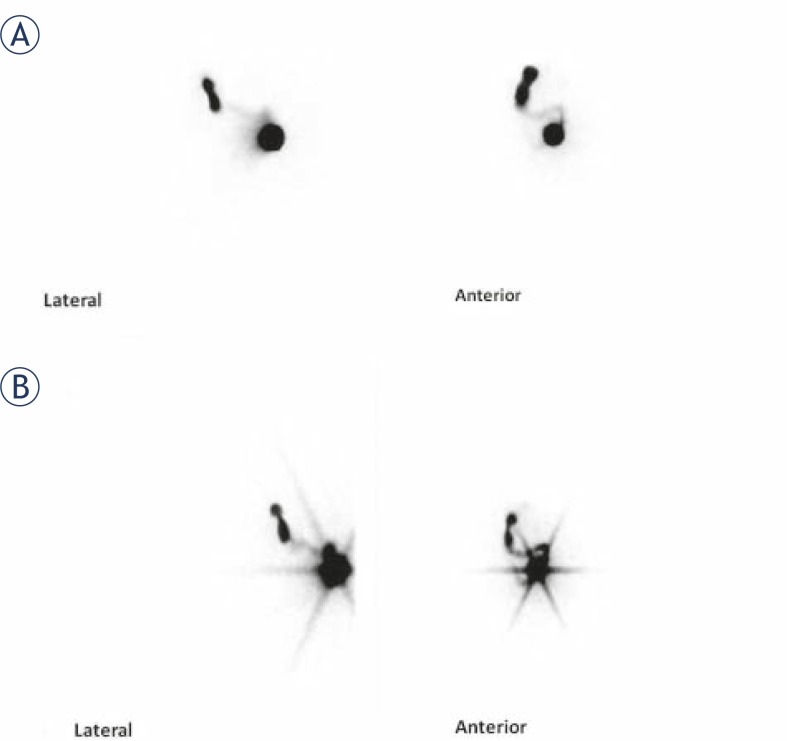
High energy (HE) collimator (A) as well as low energy high resolution collimator (B) images of the same patient. Note that two separate sentinel nodes are obvious on the low energy high resolution images. These two sentinel nodes are not shown as discrete nodes on the HE collimator images.

Table 3 shows the summary of patients’ characteristics. All images taken by LEHR collimators showed star artefact of the injection site. No image taken by HE collimator showed this effect (Figure 1). In two patients the sentinel node was visible by HE collimator but not LEHR collimator. Tumour location in both of these patients was in the upper lateral quadrant and both had history of excisional biopsy (Figure 2). In two patients, additional sentinel node was visible adjacent to the first one only on the LEHR images (Figure 3). Table 4 compares the counts of injection site as well as sentinel node(s) for HE and LEHR collimators.

## Discussion

Imaging of the axillary sentinel lymph nodes (lymphoscintigraphy) was recommended by many as a necessary part of breast cancer axillary staging using sentinel node biopsy.^13^,^14^ This imaging can be somehow challenging especially when the injection site is near the axilla which can obscure the sentinel nodes due to “shine through” and star artifacts.^7^,^15^–^17^ Sub-areolar injection of the tracer by increasing the distance between injection site and axilla can obviate this problem to some extent. However, a problem still persists especially for tumours located in the upper lateral quadrants particularly in patients with the history of previous excisional biopsy of the primary breast lesion.^17^

A method to decrease the masking effect of injection site count on the sentinel node(s) was using other types of collimators with less septal penetration.^9^ For the first time in the literature we used HE collimators for lymphoscintigraphy imaging in the current study. Our results showed better visualization of axillary sentinel lymph nodes in two patients with history of excisional biopsy and the primary tumour location in the upper lateral quadrants by HE collimator. This is most likely due to minimal septal penetration by 140 KeV photons of ^99m^Tc using the HE collimator (minimal septal penetration using HE versus 1.5% using LEHR collimators). Injection sites in these two patients were very near the axilla and resulting septal penetration masked the axillary sentinel nodes by LEHR collimator. Our findings were also supported by Tsushima *et al.* who used medium energy collimators. They reported a decrease in star-shaped artefacts with a better chance of sentinel node visualization when injection site is near the axilla.^7^

The count rate of the injection site as well as of sentinel nodes was consistently higher using HE collimator as compared to LEHR one in our study. This was most likely due to higher sensitivity of HE collimator for 140 KeV photons compared to LEHR (285.4 counts per minute per μCi for HE and 261.5 counts per minute per μCi for LEHR). This effect could also contribute to better sentinel node visualization in the above mentioned patients using HE collimators.

Star-shaped artefact which is the result of septal penetration is not the only determinant of sentinel node masking by adjacent high counts. The spatial resolution of the collimators is also of utmost importance in this regard. Several authors reported better performance of high resolution collimators for lymphoscintigraphy.^5^,^6^,^10^,^18^ We also find the same findings in two of our patients. Due to low resolution of HE collimator, the activity of the adjacent sentinel nodes were merged with each other and they can not be separable as two distinct nodes as they were on the LEHR images. Fenestra *et al.*^10^ and Lemstra *et al.*^5^ both found the same findings comparing medium energy and low energy collimators and attributed this to a higher spatial resolution. This can be especially true in our study since spatial resolution for ^99m^Tc photons (in mm at 10 cm) is 12.66 for HE and 6.64 for LEHR collimators respectively.

## Conclusions

HE collimators can be used for sentinel lymph node mapping and lymphoscintigraphy of the breast cancer patients. This collimator can almost eliminate star-shaped artefacts due to septal penetration which can be advantageous in some cases. However, to separate two adjacent sentinel nodes from each other LEHR collimators perform better.

We do not recommend routine use of HE collimators for lymphoscintigraphy due to the above mentioned low resolution. In case of significant star-shaped artefacts which can mask the sentinel nodes in the axillary area on the LEHR images, HE collimators can be helpful.

## Figures and Tables

**TABLE 1:** The specifications of high energy (HE) and low energy all purpose collimators

	**Low energy all purpose collimator**	**High energy collimator**
Hole shape	Hexagonal	Hexagonal
Number of holes (×1,000)	148	8
Hole length (mm)	24.05	50.8
Septal thickness (mm)	0.16	2
Hole diameter (mm across the flats)	1.11	3.4
Sensitivity at10 cm (count per minute/μCi) for 99mTc photons	261.5	285.4
Spatial resolution (mm at 10 cm) for 99mTc photons	6.64	12.66
Septal penetration (%) for 99m Tc photons	1.5	Almost none

**TABLE 2:** Some of the gamma camera specifications used in the current study

	**Specifications**
Field-of-view (FOV)	53.3 × 38.7 cm
Diagonal FOV	63.5 cm
Crystal	
Size	59.1 × 44.5 cm
Diagonal	69.2 cm
Thickness	9.5 cm
Photomultiplier tubes	
Total number	59
Type	Bialkali high-efficiency box-type dynodes
Array	Hexagonal
Shielding	
Back	9.5 mm
Sides	12.7 mm
Intrinsic spatial resolution	
FWHM in CFOV	≤3.8 mm
FWHM in UFOV	≤3.9 mm
Intrinsic energy resolution	≤9.9%
System spatial resolution without scatter with LEHR collimator at 10 cm	7.4 mm

FWHM = full width at half maximum; CFOV = central field of view; useful field of view UFOV; LEHR = low energy high resolution collimator

**TABLE 3:** Patients characteristics

	**Age**	**Tumour size (in cm)**	**History of excisional biopsy**	**Tumour location**	**Number of detected lymph nodes with LEHR collimator**	**Number of detected lymph nodes with HE collimator**
1	31	1.2	No	UM	1	1
2	34	2.2	No	UL	2	1
3	56	2.3	Yes	UL	0	1
4	57	2.4	No	UL	0	0
5	80	3.1	No	LL	1	1
6	43	3.6	Yes	LM	1	1
7	44	2	Yes	UL	1	1
8	36	2.1	Yes	Central	2	1
9	47	2.4	No	LL	2	1
10	58	1.3	Yes	UL	1	1
11	55	1.5	No	UL	1	1
12	45	3	Yes	LL	1	1
13	45	1.3	Yes	UM	1	1
14	78	1.4	No	UL	1	1
15	56	2.5	No	LL	1	1
16	32	2.2	No	LM	1	1
17	36	2.3	No	Central	1	1
18	45	1.7	Yes	UL	0	1
19	30	1.8	No	UL	1	1
20	34	1.9	Yes	UL	1	1

LEHR = low energy high resolution; HE = high energy; UM = upper medial quadrant; UL = upper lateral quadrant; LM = lower medial quadrant; LL = lower lateral quadrant

**TABLE 4:** Comparison of mean count rates for injection site as well as sentinel node(s) on low energy high resolution (LEHR) collimator and high energy (HE) collimators

	**High energy collimator**	**Low energy high resolution collimator**	**P value**
Injection site counts on lateral views	9127632±78784	8312982±67565	<0.001
Injection site counts on anterior views	9252343±86772	8811054±76453	<0.001
Sentinel node counts on lateral views	1799±67	1576±55	<0.0001
